# Effects of a Wild Wheat Genotype on Aphid-Parasitoid Interactions in the Geographic Center of Wheat Domestication

**DOI:** 10.3390/insects17070704

**Published:** 2026-07-07

**Authors:** Moshe Coll, Maor Elron, Eric Wajnberg

**Affiliations:** 1 Department of Entomology, The R.H. Smith Faculty of Agriculture, Food and Environment, The Hebrew University of Jerusalem, Rehovot 7610001, Israel; moshe.coll@mail.huji.ac.il (M.C.); maor.elron@gmail.com (M.E.); 2 INRAE, 400 Route des Chappes, BP 167, 06903 Sophia Antipolis Cedex, France; 3 INRIA, Projet Hephaistos, 2004 Route des Lucioles, BP 93, 06902 Sophia Antipolis Cedex, France

**Keywords:** plant domestication, wheat, aphid, parasitoid, *Rhopalosiphum padi*, *Aphidius colemani*, tri-trophic interactions

## Abstract

Domestication of wild plants to obtain agricultural cultivars is characterized by phenotypic changes that often affect herbivorous arthropods and their natural enemies. Selection for agronomically desirable traits often comes at the expense of plant defense, thus enhancing herbivore performance while hampering the activity of natural enemies. In the present study, we contrasted tri-trophic interactions on a wild and a cultivated wheat genotype, for the first time at the center of wheat domestication. Contrary to our hypothesis, a greenhouse experiment indicated that, at low aphid densities, the locally collected wild wheat genotype was more suitable for the bird cherry-oat aphid, *Rhopalosiphum padi*, than a domesticated wheat genotype. However, this was reversed at higher aphid densities; larger populations developed on the domesticated than on the wild plants in spite of a higher rate of parasitism by *Aphidius colemani* on the domesticated than the wild plants. Taken together, higher levels of biological control provided by the parasitoid may partially counteract high infestation of domesticated crops by the pestiferous aphids.

## Simple Summary

Modern farming has changed wild plants into crop varieties that produce better yields, but these changes may also affect how well crop plants resist insect pests and the effectiveness of predators and parasites of these pests. This study examined how wheat plants influence interactions between aphids that damage crops, and tiny parasitic wasps that naturally attack them. The study compared a wild wheat type collected at the region of wheat domestication (east Mediterranean) with a domesticated wheat variety. The wild wheat was expected to be better protected against aphids, but the results were mixed. When aphid numbers were low, the wild wheat actually supported more aphids than the domesticated wheat. However, when aphid numbers increased, higher aphid populations developed on the domesticated wheat than on the wild wheat. In addition, the parasitic wasps attacked more aphids on the domesticated wheat plants, helping to reduce pest numbers. The study concludes that the balance between crops, pests, and natural enemies of these pests is altered in complex ways when the tested wild plant was domesticated. These findings may help farmers and plant breeders develop wheat varieties that combine good crop production with stronger natural pest control, reducing the need for chemical pesticides and supporting more sustainable agriculture.

## 1. Introduction

The process of developing agricultural cultivars from wild plants by domestication is characterized by phenotypic changes that often affect herbivorous arthropods and their natural enemies [1]. These changes originate from both intentional and unintentional selection processes [2,3]. Desirable traits such as high yield and improved flavor often come at the expense of plant defensive abilities [4] and may be accompanied by higher nutrient composition to enhance herbivore performance further [5]. Unintentional selection may also arise from genetic linkage between traits or from random changes in plant characteristics. In addition, traits essential for plant survival in nature may lose their adaptive value in agricultural habitats [6] and may even be selected against.

These mechanisms may underlie the often-reported higher susceptibility of many crop plants to herbivore attack [7]. The increased susceptibility of domesticated plants to pests compared to that of their wild ancestors has been termed the “plant domestication-reduced defense” hypothesis [1]. A phylogenetically controlled meta-analysis confirmed that domestication did consistently reduce plant resistance to herbivores [7], but the magnitude of these effects varied across plant organs and methods for resistance measurement. In addition, domestication was not found to have consistent effects on specific plant defense traits that underlie resistance, such as secondary metabolites and physical barriers to herbivore feeding.

In the field, some plant traits may affect herbivores indirectly through complex effects on natural enemies at the third trophic level. For example, domestication has been found to alter plant morphology in ways that create refuge for pests from their parasitoids [8,9]. In some cases, however, domestication was shown to have a beneficial effect on the performance of both herbivores and their natural enemies [10,11]. For example, cultivated bean plants (*Phaseolus* spp.) were more attractive to parasitic wasps than the wild ancestral plants [12,13], and some rice cultivars were more attractive than a wild genotype to the mirid predator *Cyrtorhinus lividipennis* [14]. In contrast, Bukovinszky et al. [15] found inferior performance of aphids and their parasitoids on cultivated vs. feral *Brassica oleracea* plants. Other studies have shown that domestication may favor the herbivore but not its natural enemy [8,9,16]. Additional studies of multitrophic interactions altered by plant domestication are reviewed by Chen et al. [1] and Macfadyen and Bohan [17]. More recently, Benrey [11] discussed the various direct and indirect ways crop domestication may affect host suitability and parasitoid behavior, thus altering the nature of their interactions.

Changes in plant morphological and biochemical traits thus seem to display species-specific effects on life history traits of various arthropod species in agroecosystems [15,18,19]; these species-specific effects are expected to alter trophic interactions between herbivores and their natural enemies in various and complex ways [11].

In the present study, we used a wild and a cultivated wheat genotype to explore tri-trophic interactions in the geographic center of wheat domestication. The wild wheat genotype used for the study was collected on Mt. Carmel, Israel, the center of wheat domestication [20], and the domesticated wheat variety we used is commonly grown in this area.

The effect of wheat domestication on herbivore-parasitoid interactions has not been explored yet. Wheat (*Triticum* spp.) was first domesticated around 9000 BC in the Fertile Crescent, and wild wheat still grows in natural habitats in Israel’s central coastal plain [20]. We were able therefore to compare interactions between the native bird cherry-oat aphid (*Rhopalosiphum padi* L.) (Hemiptera: Aphididae) and its principal parasitoid wasp, *Aphidius colemani* Viereck (Hymenoptera: Ichneumonoidea: Aphidiidae) on local wild and cultivated wheat genotypes [21]. Moreover, the wild wheat genotype, the aphids and the parasitoids used in this study were all locally collected (within approximately a 10 km radius). Therefore, we studied trophic interactions among organisms with a long history of close association.

To date, studies comparing herbivore performance on wild and cultivated wheat have been focused on crop breeding. Findings indicate that primitive *Triticum* accessions are more frequently resistant [22] and less preferred by cereal aphids than modern cultivars, which were seen to be more suitable for aphid development and reproduction [23]. In maize, which is the only monocotyledon that has been studied for domestication effects on parasitoids, several parasitoid species were found to be less attracted to and have lower parasitism rates on cultivated vs. wild genotypes (reviewed in [11]). Taken together, we hypothesized that *R*. *padi* and *A*. *colemani* would be affected in different directions by wheat genotype: cultivated wheat might enhance aphid performance but reduce parasitism rates, thus exacerbating pest infestation of wheat fields.

## 2. Materials and Methods

### 2.1. Biological Materials

Seeds of wild wheat (*Triticum turgidum* ssp. *dicoccoides* (Körn.) Thell.) were provided by Y. Saranga (The Hebrew University, Rehovot, Israel). The used plants were of a single genotype originating from a natural population collected near Beit-Oren, Israel (32°42′ N 35°00′ E; see [24]). Seeds of domesticated wheat (*Triticum aestivum* L.) cv. ‘Galil’ were obtained from Hazera Genetics LTD (Brurim, Israel). Both the wild type and ‘Galil’ cv. are late-ripening. Seeds were germinated in the greenhouse for ten days in standard potting soil, and then transplanted into 1 L pots, two seedlings of either wild or domesticated wheat per pot. Pots containing pairs of 17-day-old plants were used in the experiment.

A culture of *Rhopalosiphum padi* was established from material collected from wheat fields that was maintained on wheat seedlings (cv. ‘Amit’, Hazera Genetics LTD, Brurim, Israel) in a walk-in growth chamber at 25 ± 1 °C, 16:8 L:D photoperiod.

For the experiments, *Aphidius colemani* wasps were obtained as mummified aphids from BioBee Industries (Sde-Eliyahu, Israel). BioBee wasp colonies are regularly transfused with local, field-collected material. Emerging adult wasps were kept together in a ventilated 50 mL plastic vial for 24–48 h prior to their use in the experiment; all females were assumed to have mated during this period [25].

### 2.2. Experimental Procedure

One plant in each pot was infested with six *R*. *padi* adults, in a temperature-controlled greenhouse (25 ± 3 °C) under ambient photoperiod. The other plant in each pot served at a later stage as an additional resource for the growing aphid population (see below). The infested plant was covered with a cylindrical ventilated cage (9 × 24 cm), and aphids were allowed to settle and reproduce on it for three days, after which the total number of alive aphids was recorded per plant through visual inspection. Plants harboring more than 30 aphids were used in the experiment. This ensured an excess of hosts for the ovipositing parasitoids. A total of 16 and 14 replicates of wild and domesticated wheat plants were used in the experiment, respectively.

Three days after aphid placement on the plants (day 0), a single *A*. *colemani* female was introduced into each cage through an opening at the top. Wasps were allowed to parasitize aphids for two hours, and were then removed and placed in 70% ethanol for future measurements (see below). After wasp removal, the aphids on the caged plants were monitored daily for mummy formation. On the seventh day after exposure to the parasitoids, when the infested plants began to weaken, the second plant in each pot was included in the cage and was spontaneously infested by the aphids. Wasps emerging from formed mummies were collected and stored individually in 70% ethanol. Twelve days after exposure to the parasitoids (day 12), when no additional mummies were formed, all mummies were collected into Petri dishes, allowing wasp emergence to continue. At that time, the plants were harvested at soil level, the aphids were removed and counted, and the above-ground plant parts were oven-dried at 60 °C for 24 h for dry weight determination (Sartorius CPA225D microbalance, Goettingen, Germany).

Sex determination of parasitoid offspring was done following [26]. Mummies were inspected for the presence of emergence holes. Wasp body size was determined by using the hind tibia length as a proxy; tibia length was measured using a stereo-microscope (SteREO Discovery.V8, Carl Zeiss, Oberkochen, Germany) with Zeiss ZEN 2011 Imaging Software (Carl Zeiss, Oberkochen, Germany).

### 2.3. Data Analysis

Percent parasitism was defined as the number of mummies formed divided by the number of aphids available to parasitoid attack on day 0. Parasitoid emergence rate was calculated as the number of wasp offspring divided by the number of mummies. Only replicates with more than three offspring were used for calculating the sex ratio (proportion of female offspring). The body size of wasps emerging from aphids on the two wheat genotypes was compared only for the males because of a small number of female offspring. GLM for poisson- or binomial-distributed data, with a log link or a logit function, were used for counts and percentages, respectively. When needed, repeated measures designs were used, with days taken as the repeated factor to take into account possible auto-correlation in non-independent data. For this, we used the glmmTMB package in R [27], with a first-order auto-regression model as the correlation structure. Standard ANOVA were used for normally distributed data (i.e., adult body size, dry weight, aphid density per plant biomass). All computations were done with R, version 4.4.0 [28]. Some data that did not meet ANOVA assumptions were also analyzed using non-parametric tests with distribution derived from 10,000 random permutations (MATLAB code obtained from E. Morin, The Hebrew University of Jerusalem).

## 3. Results

At the onset of the experiment, similar numbers of leaves were recorded on the wild and the domesticated plants (χ^2^ = 0.223, df = 1, *p* = 0.636). At the end of the experiment, however, the above-ground dry weight of the domesticated wheat plants was significantly higher than that of the wild plants (F_1,28_ = 66.486, *p* < 0.001; 302 ± 8 g and 179 ± 11 g, respectively).

**Figure 1:**
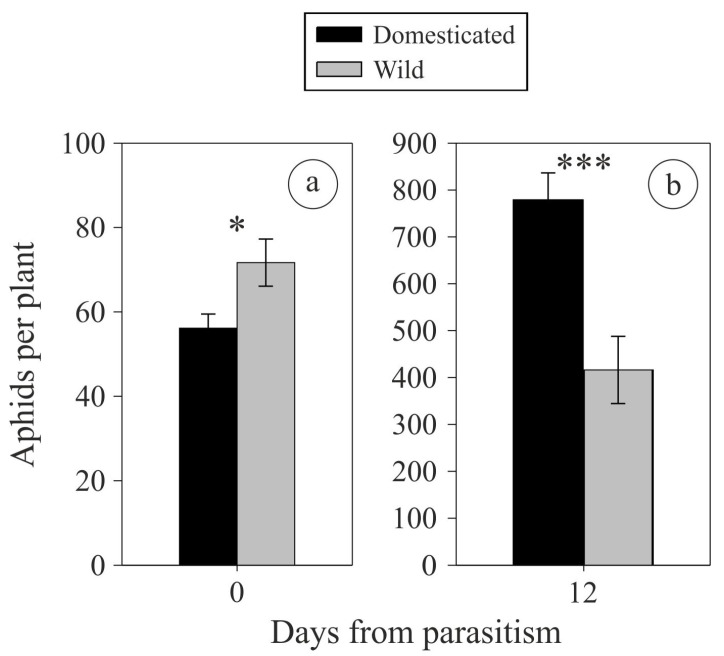
Number of aphids per plant (mean ± SE) on wild and domesticated wheat (**a**) before (day = 0) and (**b**) 12 days after parasitoid introduction for 2 h of oviposition. Three days before parasitoid introduction, each plant was infested with 6 adult aphids (* = *p* < 0.05, *** = *p* < 0.001).

Plant type had a marginally significant effect on the survival rate of the introduced adult aphids (χ^2^ = 4.109, df = 1, *p* = 0.047); slightly more adults survived on the domesticated than the wild wheat three days after they were placed on the plants. On the day of parasitoid introduction (day 0), aphid numbers were significantly higher on the wild than on the domesticated wheat (χ^2^ = 28.222, df = 1, *p* < 0.001); within three days, the aphid population increased from 6 per plant to 56.1 ± 3.32 and 71.6 ± 5.56 aphids per domesticated and wild plants, respectively (Figure 1a). Similar results were obtained when aphid numbers were expressed per plant biomass; an ANOVA shows that aphid numbers per plant biomass were affected significantly by plant type (F_1,26_ = 72.393, *p* < 0.001) as well as by plant biomass (F_1,26_ = 29.473, *p* < 0.001), but the interaction between these two factors was not statistically significant (F_1,26_ = 0.304, *p* = 0.586).

**Figure 2:**
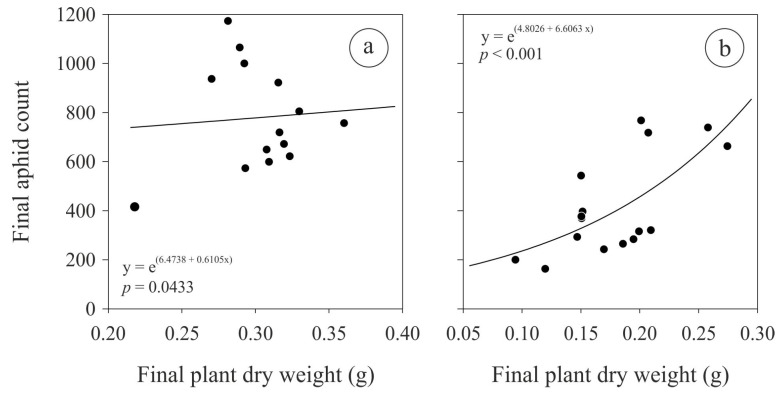
Log-linear regression model fit for the effect of plant biomass on aphid population size on (**a**) domesticated and (**b**) wild wheat plants at the end of the experiment.

In contrast to the initially higher rate of increase in aphid populations on the wild vs. domesticated wheat, an opposing trend was recorded during the following twelve days (Figure 1b). As a result, aphid numbers at the end of the experiment were significantly higher on the domesticated than on the wild plants (χ^2^ = 1684.18, df = 1, *p* < 0.001) (Figure 1b). Likewise, the final density of aphids per above-ground plant biomass was higher on domesticated than on wild plants (2601.20 ± 207.28 and 2292.65 ± 206.39 aphids per gram for domesticated and wild plants, respectively), although this difference was not statistically significant (F_1,28_ = 1.103, *p* = 0.303). The regression between final aphid population size and plant biomass was highly significant for the wild wheat (χ^2^ = 639.79, df = 1, *p* < 0.001) but only marginally significant for the domesticated plants (to χ^2^ = 4.085, df = 1, *p* = 0.0433) (Figure 2).

Mummy formation became apparent eight days after parasitism and continued for three days. Repeated measures analysis indicates that the number of mummies formed over time did not differ on wild and domesticated wheat plants (χ^2^ = 3.423, df = 1, *p* = 0.062) and that there was no significant interaction between the effects of days and plant type on the rate of mummy formation (χ^2^ = 0.104, df = 1, *p* = 0.949).

**Figure 3:**
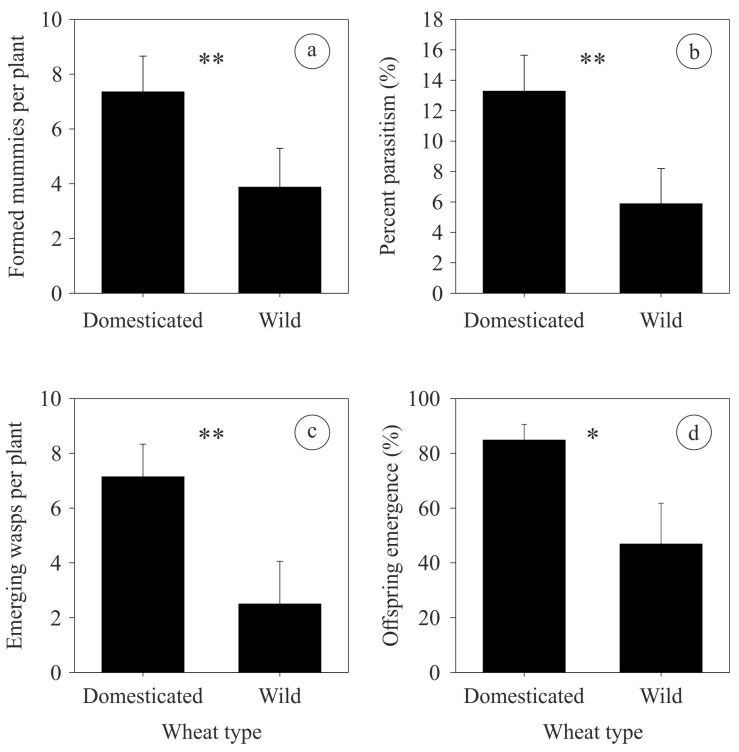
Mean (±SE) (**a**) number of formed mummies; (**b**) percent parasitism; (**c**) number of emerging wasps; and (**d**) percent of parasitoid offspring emergence from the mummies, as a result of a two-hour foraging by a single *A*. *colemani* female, parasitizing *R*. *padi* aphids on wild or domesticated wheat plants (* = *p* < 0.05; ** = *p* < 0.01).

Whereas no mummies were formed on 31.25% (5 out of 16) of the wild wheat plants, this variable was only 7.14% (1 out of 14) for the domesticated plants. Thus, twice as many mummies were formed per plant on the domesticated than on the wild wheat (Figure 3a). The influence of plant type on the number of mummies was significant also when the number of aphids available to the parasitoid on day = 0 served as a covariate (χ^2^ = 16.505, df = 1, *p* < 0.001); aphid number and plant type did not interact in their effects on mummy formation (χ^2^ = 1.239, df = 1, *p* = 0.266). Because more aphids were available on day 0 to the ovipositing wasps on wild than domesticated plants (Figure 1), percent parasitism was significantly lower on the former than the latter plants (χ^2^ = 34.688, df = 1, *p* < 0.001, Figure 3b).

**Figure 4:**
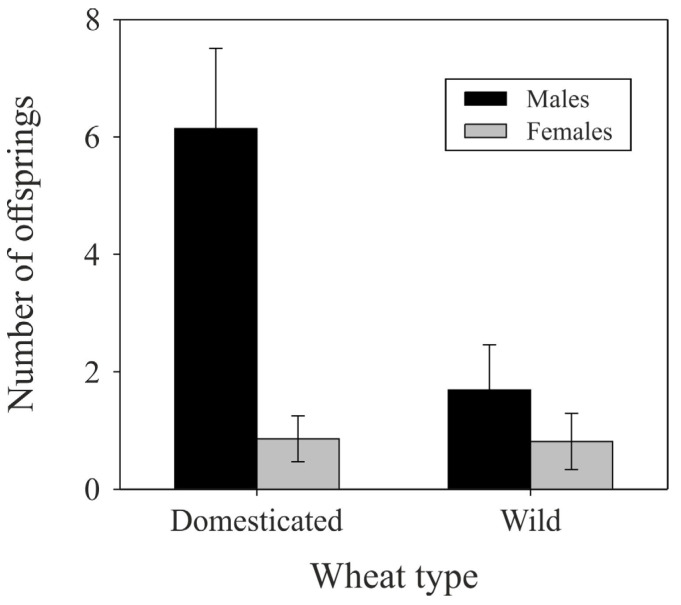
Mean number (±SE) of male and female parasitoid offspring produced per ovipositing female on domesticated and wild wheat plants.

The number of wasp offspring emerging from aphids on domesticated wheat was 2.7 times higher than from aphids on wild wheat (χ^2^ = 35.201, df = 1, *p* < 0.001, Figure 3c). On the domesticated wheat, 85.3% of the mummies yielded adult parasitoids, whereas only 53.2% produced adults on the wild wheat (*p* = 0.020; random permutations, Figure 3d). The total number of female offspring emerging per plant did not differ significantly between wheat types (χ^2^ = 0.018, df = 1, *p* = 0.894). However, significantly more males emerged from aphids on the domesticated than the wild genotype (χ^2^ = 40.766, df = 1, *p* < 0.001, Figure 4). Finally, a higher proportion of ovipositing wasps failed to produce female offspring on domesticated than on wild wheat (61.5% and 50%, respectively). Overall, the effective proportion of female offspring differed significantly between wild and domesticated wheat (0.33 ± 0.12 and 0.11 ± 0.04, respectively; χ^2^ = 7.272, df = 1, *p* = 0.007).

**Figure 5:**
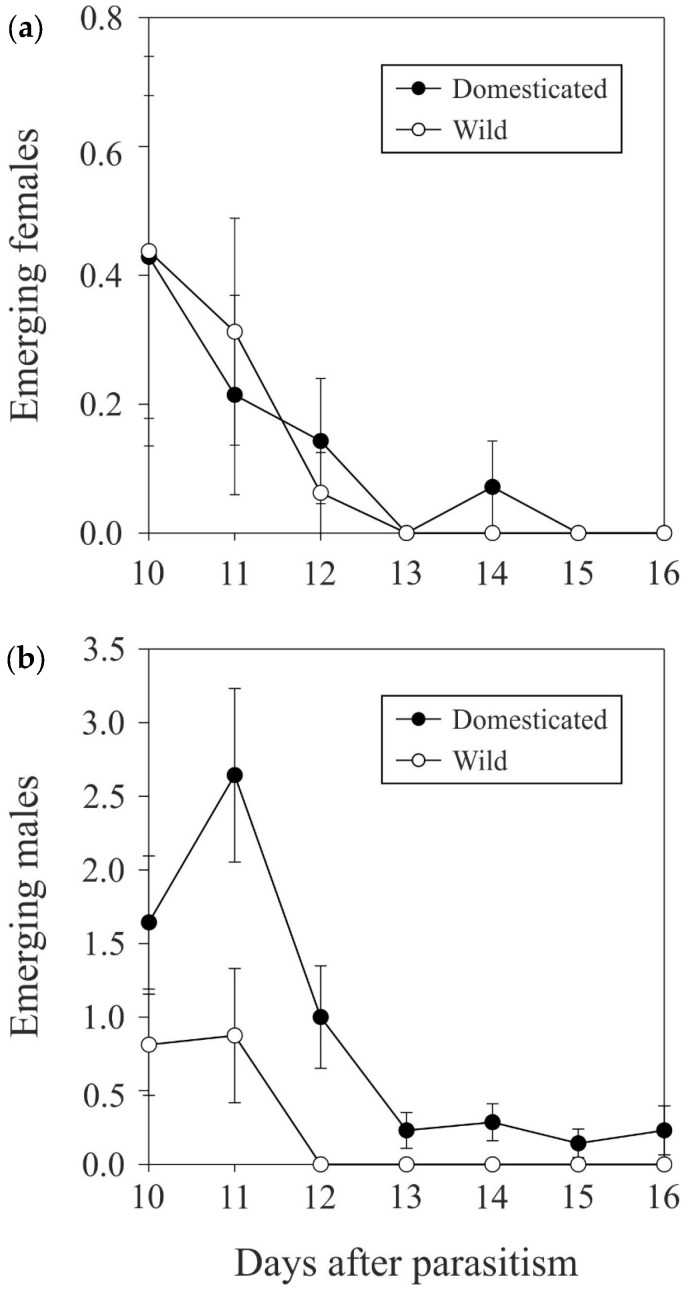
Daily mean number (± SE) of emerging female (**a**) and male (**b**) parasitoids per plant on domesticated and wild wheat plants, 10 to 16 days after parasitism.

On day twelve after parasitism, no additional wasps emerged from mummies on the wild wheat; wasps continued to emerge on the domesticated wheat for four more days (Figure 5). Repeated measures log-linear regression showed that female wasps emergence pattern did not differ significantly between domesticated and wild wheat (χ^2^ = 0.215, df = 1, *p* = 0.643, Figure 5a), the day of emergence did not influence female emergence rate (χ^2^ = 2.236, df = 6, *p* = 0.897), and there was no significant interaction between plant type and emergence day (χ^2^ = 0.754, df = 6, *p* = 0.993). Yet, it seems that plant type and emergence day may have an effect on male emergence pattern (Figure 5b). However, the low number of emerging males precluded statistical analysis. It seems therefore that the recorded differences in wasp emergence on the two plant types (Figure 4) are due to fewer males emerging on wild than on domesticated plants.

Offspring body size was independent of mother body size (R^2^ = 0.003, *p* = 0.691). Whereas the body size of female offspring did not differ between wheat genotypes (F_1,22_ = 0.009, *p* = 0.927; 0.46 ± 0.01 and 0.46 ± 0.01 mm, on domesticated and wild plants, respectively), male offspring originating from aphids reared on wild wheat were significantly larger than those developing on the domesticated wheat (0.43 ± 0.01 and 0.40 ± 0.01 mm, respectively; F_1,16_ = 6.472, *p* = 0.022). A negative relationship was found between emergence day and male body size (mean per mother) during the first four days of emergence on both the domesticated and the wild wheat (R^2^ = 0.371, *p* < 0.001 and R^2^ = 0.523, *p* = 0.012, respectively); larger males emerged earlier, during peak male emergence time, than later-on (0.43 ± 0.01 and 0.37 ± 0.01 mm on days 10–11 and 13–16 after parasitism, respectively; F_1,83_ = 21.819, *p* < 0.001).

## 4. Discussion

Contrary to our hypothesis and experimental evidence from other studies [7], the wild wheat genotype tested in the current study appears to be more suitable for *R*. *padi* than the domesticated wheat. This is evident from the differential rate of increase in aphid populations during the first three days of the experiment. During that time, aphid populations were not constrained by host plant availability (i.e., aphid crowding) or parasitoid attack. This result concerning the generalist *R*. *padi* is also in contrast to the suggestion that plant domestication is more beneficial for generalist herbivores than for specialists [29].

The obtained data show that the higher rate of aphid population growth on wild plants was not due to the differential effect of the plants on the survival of founding adult aphid because somewhat more adult aphids survived on the domesticated than wild plants three days after being placed on the plants. These results could therefore be attributed primarily to plant suitability for aphid reproduction, development or survival, or any combination of these fitness components. Later in the experiment, when aphid density was high, population growth appeared to be more limited on the more suitable wild host plants. As a result, and in spite of a higher parasitism on the domesticated plants, more aphids inhabited the domesticated than the wild wheat at the end of the experiment. However, aphid density (numbers per plant biomass) at the end of the experiment did not differ significantly between domesticated and wild plants. In addition, a strong correlation was found between the final population size and plant biomass, but only for the wild wheat. Taken together, these results, and the higher parasitism on domesticated plants, suggest that aphid populations may be constrained by different density-dependent mechanisms on the two host genotypes in our experimental system; aphid growth was likely limited by intraspecific competition on the wild wheat, and by parasitoid activity on the domesticated wheat.

In contrast to aphid response, parasitoid performance was poorer on the wild host than on the domesticated wheat plants; fewer mummies were formed on the wild plants, and many of these plants did not yield mummies at all. It is unlikely that aphid availability was a limiting factor for ovipositing parasitoids in the experiment, because percent parasitism remained low and results were unaffected when aphid density was used as a covariate. In addition, the lower rate of emergence of adult wasps from mummies formed on wild than on domesticated wheat may be indicative of inferior-quality hosts on the former plants. It therefore appears that both variation in parasitoid behavior and differential survival of wasp offspring underlie differences in the recorded rates of parasitism on the two plant types. Further investigation is needed to distinguish the relative magnitude of the two suggested effects of the wild genotype on the parasitoid.

Protandry, in which males emerge prior to females, is common among parasitoids [30], including in some *Aphidius* species [31]. In the present study, however, the emergence of male and female parasitoid offspring was fairly synchronized on the wild wheat, whereas males continued to emerge for four additional days on the domesticated wheat. Female parasitoids tend to preferentially oviposit in the largest available hosts [15,32]. The late emergence of males on the domesticated wheat could be attributed to slower development on lower quality, smaller hosts (e.g., [33]). Indeed, the later-emerging males were relatively small.

Although overall, parasitism on the wild wheat was lower than that on the domesticated wheat, a higher proportion of female parasitoids were produced on the former plants. *A. colemani* reared on *R*. *padi* is known to have a male-biased sex ratio [33]. The more female-biased sex ratio in the wild vs. the domesticated wheat could have arisen from differential sex allocation by ovipositing wasps, from differences in parasitoid survival within the host, or both. Parasitic Hymenoptera tend to deposit female eggs in larger hosts [15,32], and aphid body size has been shown to affect host selection and parasitoid performance (e.g., [34,35]). Therefore, a male-biased sex ratio is expected when large hosts are scarce, which is consistent with the lower suitability of the domesticated plants for aphids. Nonetheless, the possibility for differential mortality of male and female wasp offspring developing within aphids on different host plants could not be ruled out. Finally, the obtained results could not be attributed solely to smaller male body size, because female body size could not be compared due to the small sample size.

Our results appear to support the hypothesis that wild plants are less suitable for parasitoid development than their domesticated relatives. In contrast, the aphids in this study exhibited faster population growth on the wild genotype compared to the domesticated plants. Taken together, the higher levels of biological control provided by the parasitoid on the cultivated genotype may partially compensate for the higher infestation of these plants by the aphids. Furthermore, results highlight the variability in the effects of plant domestication on pests and their natural enemies and thus the need for a case-by-case examination. These conclusions should, however, be treated with caution as only one wild and one domesticated genotype were used in this study, and in light of the relatively small number of replications. Nonetheless, the two selected genotypes co-evolved within the same geographic region, therefore increasing the validity of the investigated trophic interactions. Also, the study yielded statistically significant results in spite of the seemingly small sample sizes. Finally, the obtained results are in agreement with a companion field study that compared arthropod community structure and function on the same wild and cultivated genotypes, even in the presence of numerous other aphid and parasitoid species on these wheat genotypes in the field [21].

## Data Availability

Data of the research presented here are available on figshare with the following DOI reference: https://doi.org/10.6084/m9.figshare.29364137.
